# RUNX1 regulates MCM2/CDC20 to promote COAD progression modified by deubiquitination of USP31

**DOI:** 10.1038/s41598-024-64726-w

**Published:** 2024-06-17

**Authors:** Wei Tian, Jingyuan Zhao, Xinyu Zhang, Pengfei Li, Xuening Li, Yuan Hong, Shuai Li

**Affiliations:** 1 The First Affiliated Hospital of Dalian Medical University, Dalian, China; 2 Stem Cell Clinical Research Center, The First Affiliated Hospital of Dalian Medical University, Dalian, China; 3 Clinical Laboratory Center, Dalian Municipal Central Hospital, Dalian, China; 4 Dalian Medical University, Dalian, China; 5 Department of Pharmacy, The First Affiliated Hospital of Dalian Medical University, Dalian, China

**Keywords:** Colon adenocarcinoma, RUNX1, USP31, Transcription factors, Prognosis, Colorectal cancer, Colorectal cancer

## Abstract

Colon adenocarcinoma (COAD) is the second leading cause of cancer death, and there is still a lack of diagnostic biomarkers and therapeutic targets. In this study, bioinformatics analysis of the TCGA database was used to obtain RUNX1, a gene with prognostic value in COAD. RUNX1 plays an important role in many malignancies, and its molecular regulatory mechanisms in COAD remain to be fully understood. To explore the physiological role of RUNX1, we performed functional analyses, such as CCK-8, colony formation and migration assays. In addition, we investigated the underlying mechanisms using transcriptome sequencing and chromatin immunoprecipitation assays. RUNX1 is highly expressed in COAD patients and significantly correlates with survival. Silencing of RUNX1 significantly slowed down the proliferation and migratory capacity of COAD cells. Furthermore, we demonstrate that CDC20 and MCM2 may be target genes of RUNX1, and that RUNX1 may be physically linked to the deubiquitinating enzyme USP31, which mediates the upregulation of RUNX1 protein to promote transcriptional function. Our results may provide new insights into the mechanism of action of RUNX1 in COAD and reveal potential therapeutic targets for this disease.

## Introduction

Colon adenocarcinoma (COAD) is a common malignant tumor in the gastrointestinal tract and is the second leading cause of death from cancer^1^, the incidence among young people is increasing year by year^2^. The early symptoms of COAD are hidden, and most of the patients are in the middle and late stages, so it is difficult to treat. Although the diagnosis and treatment of COAD have made significant progress, the curative effect and prognosis are still not optimistic^3–5^. There is still a need to explore the pathogenesis of COAD and provide new strategies for targeted treatment of COAD.

Transcription factors are an important class of molecules that intracellularly regulate the transcriptional state of specific genes and are involved in the process of tumorigenesis development^6,7^. Runt-related transcription factor 1 (RUNX1) is a member of the RUNX family of transcription factors (RUNX1, RUNX2 and RUNX3)^8^. The RUNX transcription factors family is an important regulator of many developmental processes, including cell proliferation, differentiation, apoptosis and other important biological processes^9^. Moreover, cancer cell metastasis, proliferation and cancer stemness are a few of the important processes that RUNX1-mediated signaling pathways regulate^10^. Previous studies have suggested that RUNX1 may be associated with the development of cancers such as acute leukemia, renal cell, colorectal, breast, uterine and ovarian adenocarcinomas^11–14^. Although the understanding of the role of RUNX1 in many malignancies, the molecular mechanisms of its regulation in COAD remain to be fully investigated.

Ubiquitination is a modification at the protein level where ubiquitin binds to the target protein via multiple ubiquitin ligases and degrades to regulate the protein expression level^15,16^. In contrast, deubiquitination is responsible for the removal of ubiquitin/polyubiquitin chains from protein substrates and reverses the degradation of ubiquitination^16^. Transcription factors as a protein can be modified by ubiquitination, which controls transcription factor activity and achieves a shift in transcription factor activity to complete transcriptional activation of related genes. Studies on the regulatory mechanisms of ubiquitination modifications in transcription factors will contribute to the regulation of oncogene transcription and the treatment of COAD^17–19^.

In this study, we explored potential diagnostic and prognostic biomarkers in COAD through comprehensive bioinformatics analysis of TCGA data, and finally identified RUNX1 as a biomarker, and the overexpression of RUNX1 was detrimental to the prognosis and survival of COAD patients. We also elucidated the role and molecular mechanism of RUNX1 in COAD by cell proliferation assay, transcriptome sequencing, chromatin immunoprecipitation, and ubiquitination analysis experiments.

## Result

### Construction of transcriptional prognostic models based on screened transcription factors

**Figure 1:**
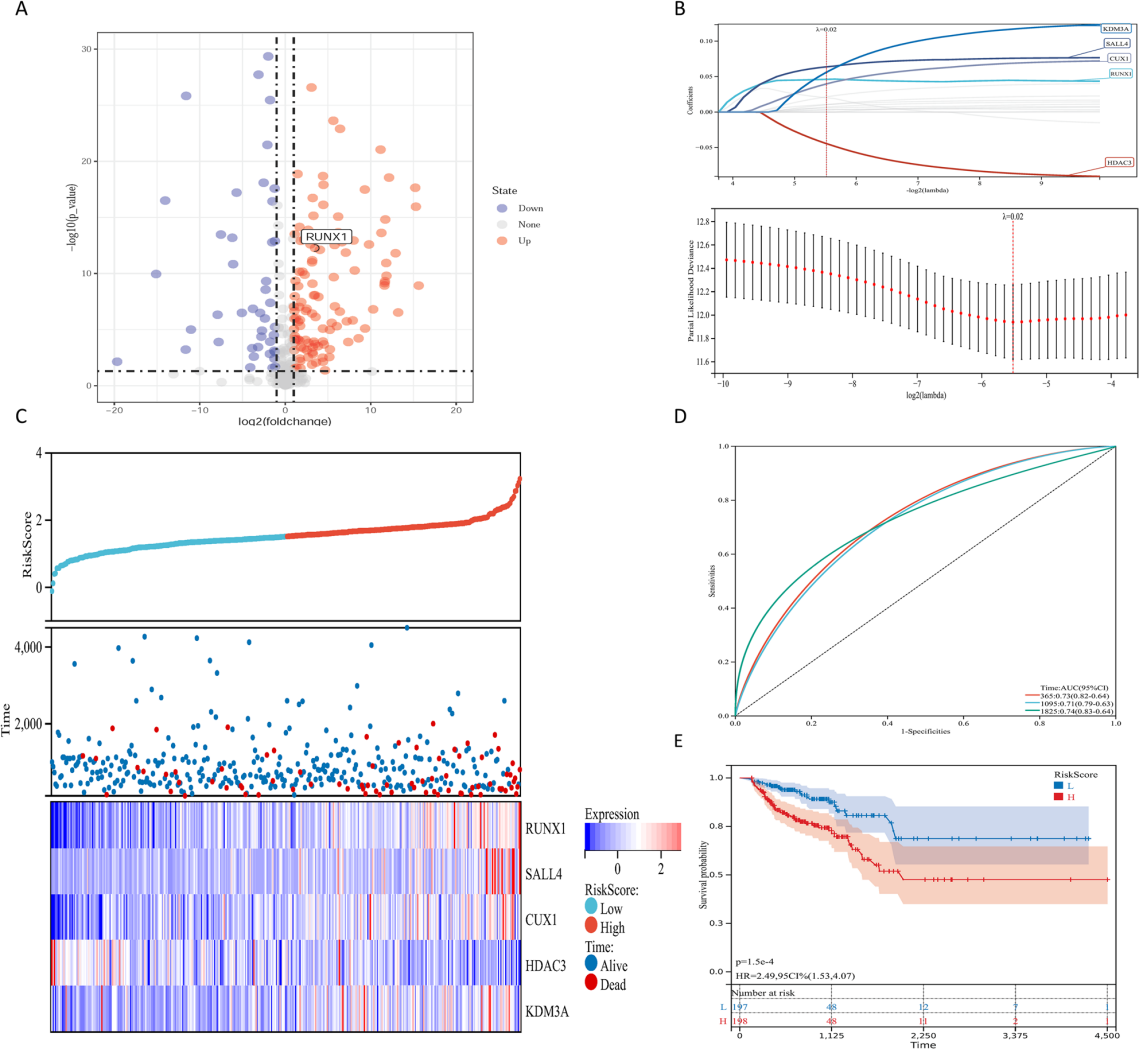
Construction of transcriptional prognostic models based on screened transcription factors. (**A**) Volcano plot of 129 TFs matched with DEGs obtained from the COAD gene expression profile of the TCGA database. (**B**) Upper, LASSO coefficients profiles of key OS-related TFs. Lower, LASSO regression with tenfold cross-validation obtained 5 prognostic genes using minimum lambda value. (**C**) Upper, the curve of risk score. Middle, survival status of the patients. More dead patients corresponding to the higher risk score. Lower, heatmap of the expression profiles of the five prognostic genes in low- and high-risk group. (**D**) Time-dependent ROC analysis the of the five-gene signature. ROC receiver operating characteristic. (**E**) Kaplan–Meier survival analysis of the five-gene signature.

We obtained gene expression profiles from TCGA, we identified transcription factors, 101 TFs were identified as significantly different by matching TFs to DEGs. Among these, 63 TFs were upregulated (Fig. 1A). LASSO regression analysis was used to select the key OS-related TFs for modeling (Fig. 1B). As a result, a total of five TFs (CUX1, HDAC3, KDM3A, SALL4, and RUNX1) were identified and selected to develop a prognostic signature. According to the median risk score, patients were divided into high-risk and low-risk groups (Fig. 1C). As the risk score of patients increased, the number of deaths increased (Fig. 1C). The AUC of our signature for 1-year, and 3-year and 5-year OS were 0.73, 0.71 and 0.74, respectively, indicating the high predictive capacity of the signature (Fig. 1D). The Kaplan–Meier analysis showed that patients in the high-risk group had significantly shorter OS than patients in the low-risk group (Fig. 1E).

### RUNX1 is upregulated in COAD and correlates with a poor prognosis

**Figure 2:**
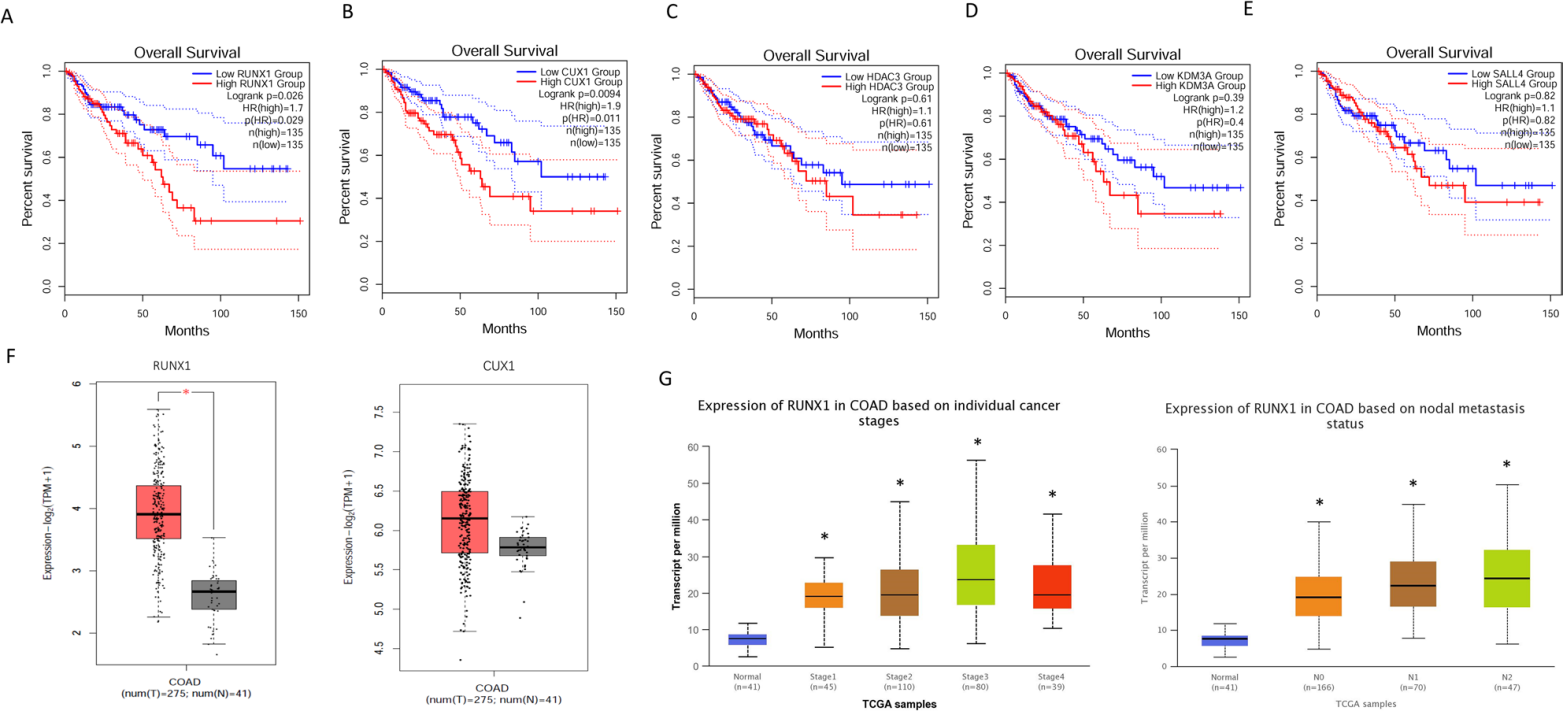
RUNX1 is upregulated in COAD and correlates with a poor prognosis. (**A**-**E**) Provided by GEPIA, the effect of CUX1, HDAC3, KDM3A, SALL4 and RUNX1 gene expression on survival. (**F**) Expression of RUNX1 and CUX1 in COAD tissues and normal tissues. *P < 0.05. (**G**) Expression of RUNX1 in COAD based on individual cancer stages and nodal metastasis status. *P < 0.05.

GEPIA was used to validate the expression and impact on survival of these prognosis-related genes, CUX1, HDAC3, KDM3A, SALL4, and RUNX1 in COAD (Fig. 2A-E). The results confirmed that patients with the RUNX1 gene high expression group had a worse prognosis (Fig. 2A). Subsequently, we analyzed the expression levels of RUNX1 and CUX1 in COAD tissues compared to normal tissues (Fig. 2F). We found that RUNX1 was significantly upregulated in COAD, while there was no apparent trend in the expression of CUX1. Therefore, RUNX1 was selected as the focus of our subsequent investigations. First, we focused on the difference of RUNX1 gene expression among patients with different stages of COAD. We found that stage 3 with the higher the expression of RUNX1 (Fig. 2G). In terms of the direction of lymph node metastasis, the more serious the degree of COAD metastasis, the more lymph are odes involved, and the higher the expression level of RUNX1 (Fig. 2G). These results indicated that RUNX1 is upregulated in COAD and associated with a poor prognosis. RUNX1 showed up-regulated expression in a variety of cancers in pan-cancer analysis (Supplementary Fig. 1), and it may be a gene with broad oncogenic ability.

### Downregulation of RUNX1 inhibits the growth and migration of COAD cells

**Figure 3:**
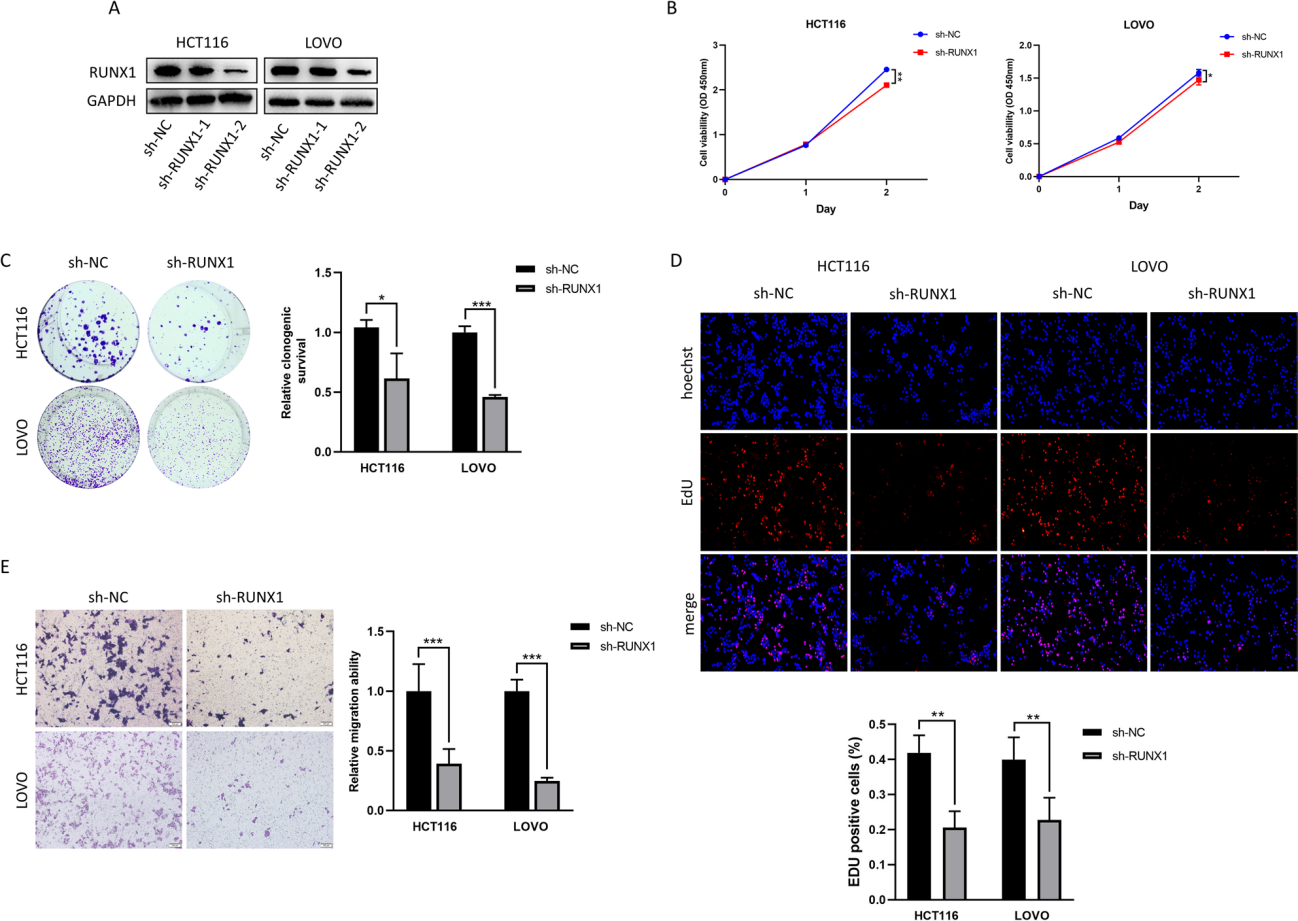
Downregulation of RUNX1 inhibits the growth and migration of COAD cells. (**A**) Levels of RUNX1 protein in HCT116 and LOVO cells transfected with a plasmid encoding shRUNX1 were analyzed by Western blotting (Original blots are presented in Supplementary Fig. 7). (**B**) Cell proliferation assays of HCT116 and LOVO cells transfected as described above at two time points of 24 and 48 h. *P < 0.05, **P < 0.01. (**C**-**E**) Colony formation, EdU staining and transwell assays of HCT116 and LOVO cells transfected as above. *P < 0.05, **P < 0.01, ***P < 0.001.

To study the biological function of RUNX1 in COAD, we established RUNX1-silenced cells using a plasmid vector carrying shRNA in HCT116 and LOVO cells. Compared with the control group, the experimental results demonstrate a significant reduction in the expression level of RUNX1 protein in the treatment group with the construct sh-RUNX1-2 (Fig. 3A), and the construct sh-RUNX1-2 was used for subsequent experiments. In cells with RUNX1 knockdown compared to control cells, the growth ability of the cells was much lower (Fig. 3B-D). Furthermore, transwell migration results demonstrated that RUNX1 knockdown dramatically reduced the capacity for COAD cells to migrate (Fig. 3E). Overall, our findings suggested that RUNX1 down-expression might prevent COAD cell proliferation and metastasis.

### RUNX1 increased the expression of CDC20 and MCM2

**Figure 4:**
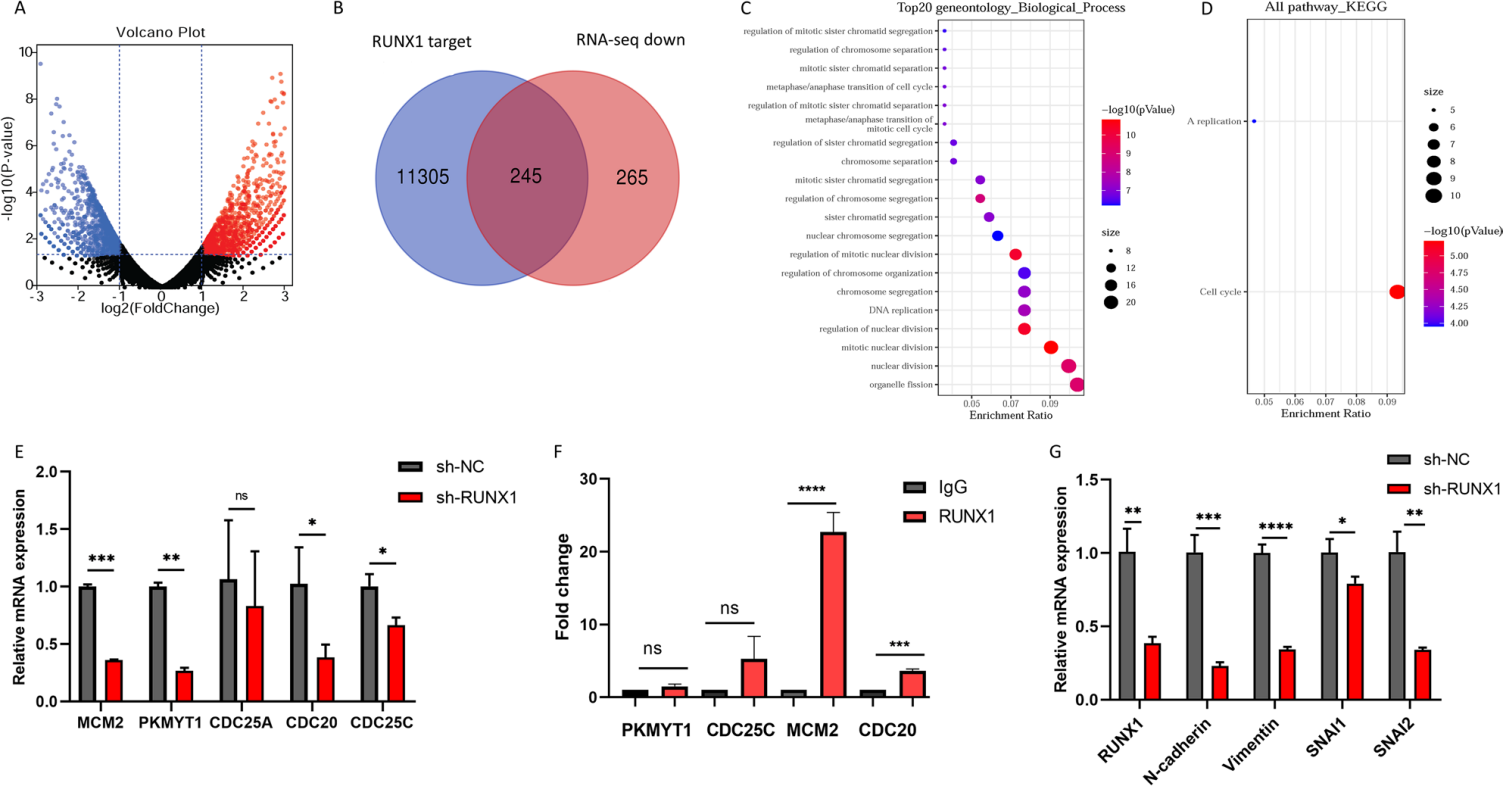
RUNX1 increased the expression of CDC20 and MCM2. (**A**) Volcano plot of differentially expressed genes in COAD compared to normal tissue. Red nodes represent genes that are obviously up-regulated. Blue nodes represent genes that are obviously down-regulated. (**B**) Genes shared by RUNX1 targets and RNA-seq down-regulated genes. (**C**) Significantly enriched KEGG pathways of the above obtained genes. (**D**) Significantly enriched GO biological process of the above obtained genes. (**E**) The expression levels of PKMYT1, CDC25C, MCM2, CDC25A, CDC20 by real-time PCR in HCT116 cells. *P < 0.05, **P < 0.01, ***P < 0.001. (**F**) The binding of RUNX1 to CDC20 and MCM2 could be verified by ChIP-qPCR in HCT116 cells. ***P < 0.001, ****P < 0.0001. (**G**) The expression levels of N-cadherin, Vimentin, SNAI1, and SNAI2 by real-time PCR in HCT116 cells. *P < 0.05, **P < 0.01, ***P < 0.001, ****P < 0.0001.

In order to find the target gene for RUNX1 transcription, transcriptome sequencing was performed after knockdown of RUNX1. The overall distribution of the DEGs can be represented by a volcano plot (Fig. 4A). 510 down-regulated genes were found.

Silencing the RUNX1 gene repressed its transcriptional function, resulting in the down-regulation of target genes. The hTFtarget has integrated thousands of ChIP-seq datasets to predict reliable TF-target regulations. 11,550 transcribed genes of RUNX1 were obtained by hTFtarget database. We obtained 245 overlapping genes (Fig. 4B) that may be transcribed genes of RUNX1. These genes were subjected to gene enrichment analysis, GO analysis showed that the biological processes of these target genes were concentrated in mitosis and organelle division (Fig. 4C), and cellular component of these target genes were chromosomal region (Supplementary Fig. 2). The KEGG analysis showed that these target genes were concentrated in the cell cycle pathway (Fig. 4D).

The results of the enrichment analysis showed that CDC45, PKMYT1, CDC25C, MCM3, MCM2, CDC25A, CDC20, MCM7, PLK1, BUB1B were enriched in the cell cycle pathway. Therefore, we verified the expression levels of the genes by real-time PCR (Fig. 4E). The results showed that compare with control, RUNX1 knockdown down-regulated the expression of CDC20 and MCM2 genes which promote cell cycle progression. Then CHIP-qPCR experiments were performed on the enriched genes, and the results showed a significant difference in the enrichment efficiency of MCM2 and CDC20 (Fig. 4F). According to the results, MCM2 and CDC20 might serve as the target genes for RUNX1 transcription. CDC20 and MCM2 expression was upregulated in COAD compared with normal tissues (Supplementary Fig. 3), which may be caused by transcription of CDC20 and MCM2 by RUNX1 in COAD. In COAD tissues, RUNX1 was closely associated with the epithelial-mesenchymal transition (EMT) pathway, and CDC20 and MCM2 were closely associated with the cell cycle pathway (Supplementary Fig. 4). Previous findings showed a positive and statistically significant correlation between the expression of RUNX1 and several molecules associated with EMT, including N-cadherin, Vimentin, and Snail^20^. Therefore, for further validation, we examined the expression levels of EMT-related molecules after RUNX1 knockdown (Fig. 4G). The results showed that the expression levels of N-cadherin, Vimentin, SNAI1, and SNAI2 were significantly reduced after knockdown of RUNX1. This result that is consistent with our experimental results above, RUNX1 increased the migration and invasion ability of COAD cell lines, and this effect was associated with EMT.

### USP31 as a deubiquitinating enzyme is physically associated with RUNX1

Next, we continued to investigate the factors that influence the role of RUNX1 protein in COAD. Ubiquitination and deubiquitination are two important modalities of protein post-translational modifications that play an important role in regulating protein stability. The ubiquitin-specific protease (USP) family affects protein deubiquitination and plays a key role in regulating protein levels^21^. To investigate whether USP plays a role in COAD to influence RUNX1 levels, we first analyzed the expression of USP in COAD.

**Figure 5:**
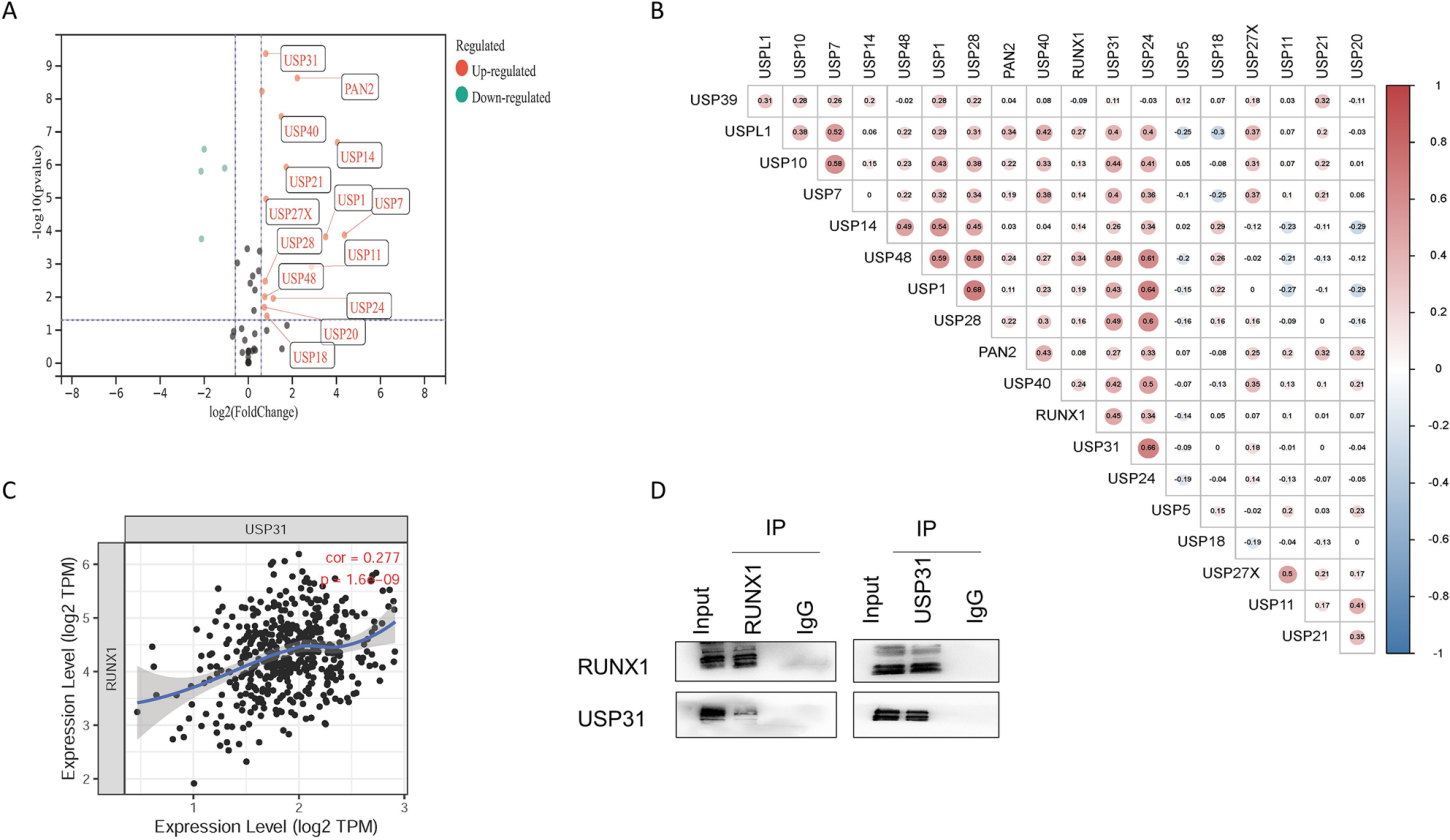
USP31 as a deubiquitinating enzyme is physically associated with RUNX1. (**A**) Volcano plot of differentially expressed genes of the USP family in COAD compared to normal tissue. Red nodes represent genes that are significantly up-regulated. Green nodes represent significantly down-regulated genes. (**B**) Matrix diagram of the expression correlation of 17 genes in the USP family with RUNX1. The size of the bubbles represents the correlation level. The color of the bubbles represents the FDR level. Red: Positive correlation. Blue: negative correlation. (**C**) Fitted curves obtained from the TIMER database for the expression correlation between RUNX1 and USP31 in COAD (n = 458). (**D**) Coimmunoprecipitation (co-IP) of RUNX1 and USP31 from HCT116 cells. IgG was used as the control (Original blots are presented in Supplementary Fig. 8).

After analyzing the differences in USP expression in COAD, it was found that most USP genes were up-regulated in TCGA COAD (Fig. 5A). Further analysis of the correlation between up-regulated USP genes and RUNX1 expression (Fig. 5B), and we found that the USP31 expression level was related to the RUNX1 expression fitting curve (Fig. 5C). To verify whether the two proteins interact with each other, through co-IP, and the results show that RUNX1 can bind to USP31 (Fig. 5D). This suggested that USP31, a deubiquitinating enzyme, is physically linked to RUNX1.

### USP31 mediates the deubiquitination of RUNX1 and regulates its transcriptional function

**Figure 6:**
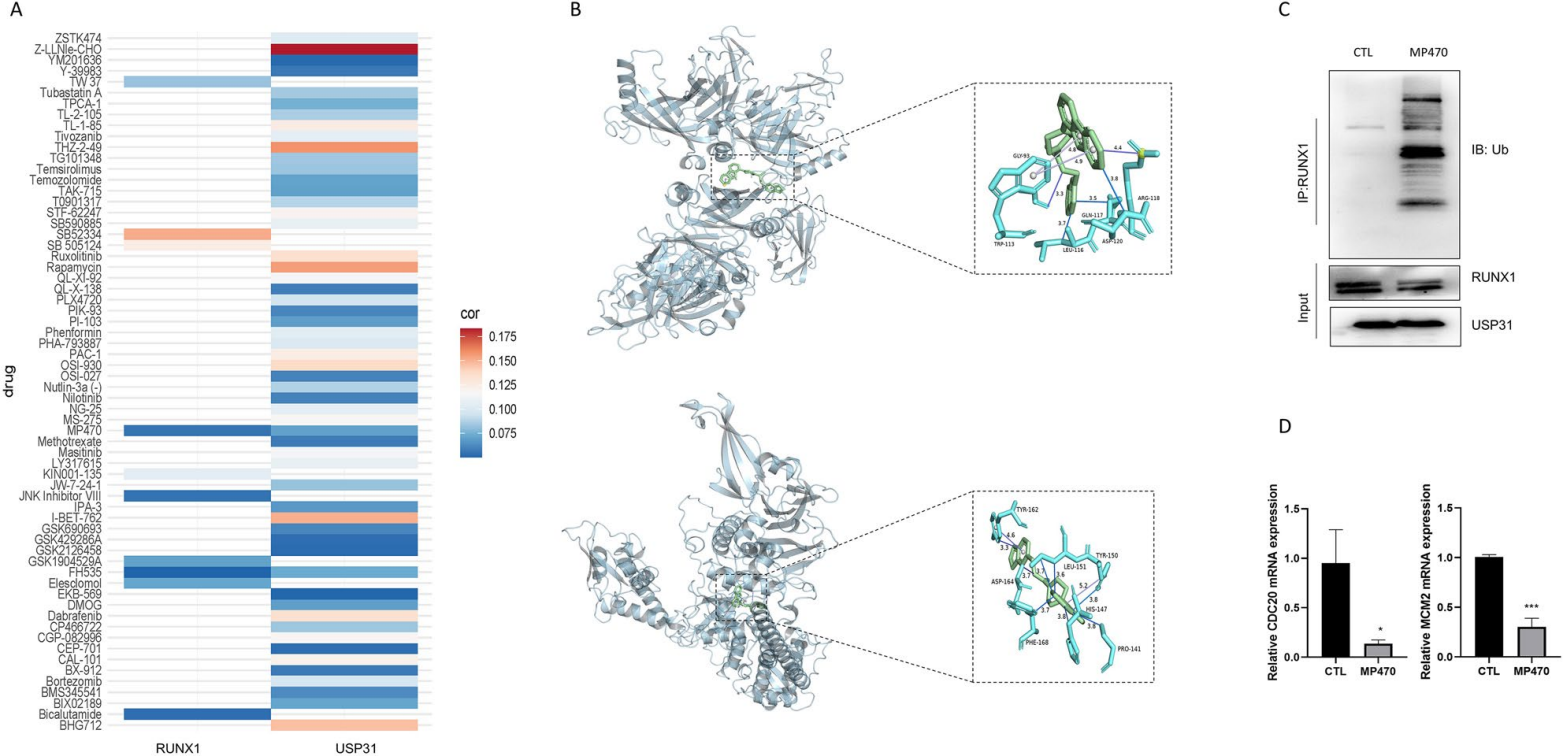
Deubiquitinase function is required for USP31-mediated activation of RUNX1. (**A**) Heat map of 64 small molecule inhibitors associated with RUNX1 and USP31. (**B**) Binding mode of MP470 to RUNX1 and USP31 by molecular docking (the two results with the lowest binding energy). Upper, Molecular docking results of MP470 and RUNX1 (binding energy − 6.103 kcal/mol). Lower, Molecular docking results of MP470 and USP31 (binding energy − 8.651 kcal/mol). (**C**) Co-IP from untreated and MP470-treated HCT116 cells. IgG was used as the control (Original blots are presented in Supplementary Fig. 9). (**D**) The expression levels of CDC20 and MCM2 by real-time PCR in HCT116 cells after MP470 treatment. *P < 0.05, ***P < 0.001.

As RUNX1 can bind to USP31, to further investigate the interaction by searching for inhibitors against both by Genomics of Drug Sensitibity in Cancer (GDSC). We analyzed the most relevant small molecule inhibitors of RUNX1 and USP31 (Fig. 6A). Two inhibitors, MP470 and FH535 were found to act on both RUNX1 and USP31. To assess the affinity of the drug candidates for their targets, we performed a molecular docking analysis. Following the molecular docking steps described in the methods section, we molecularly docked MP470 and FH535 to RUNX1 protein and USP31 protein, respectively. And the results showed that the binding energy of FH535 to RUNX1 was less than − 5.0 kcal/mol (Supplementary Table 1), indicating a poor affinity and unstable binding. However, the binding energy of MP470 to both proteins was less than − 5.0 kcal/mol (Supplementary Table 3), indicating better binding activity. The docking results with the lowest binding energy are shown in Fig. 6B. Therefore, we selected MP470 for interfering with the interaction between RUNX1 and USP31. We treated HCT116 cells with MP470 (0.1 µM, 24 h) for ubiquitination analysis and detected ubiquitination levels by Western blot (Fig. 6C). The results showed that MP470 increased the ubiquitination level of RUNX1, leading to a decrease in RUNX1 protein level, which may be due to its inhibition of the interaction between RUNX1 and USP31. Real-time PCR of the cells revealed that MP470 treatment leading to ubiquitination also inhibited RUNX1 transcription of CDC20 and MCM2 expression (Fig. 6D). Taken together, these data further supported that the binding of USP31 to RUNX1, and showed that USP31 could mediate the deubiquitination of RUNX1 and regulate its transcriptional function.

## Discussion

**Figure 7:**
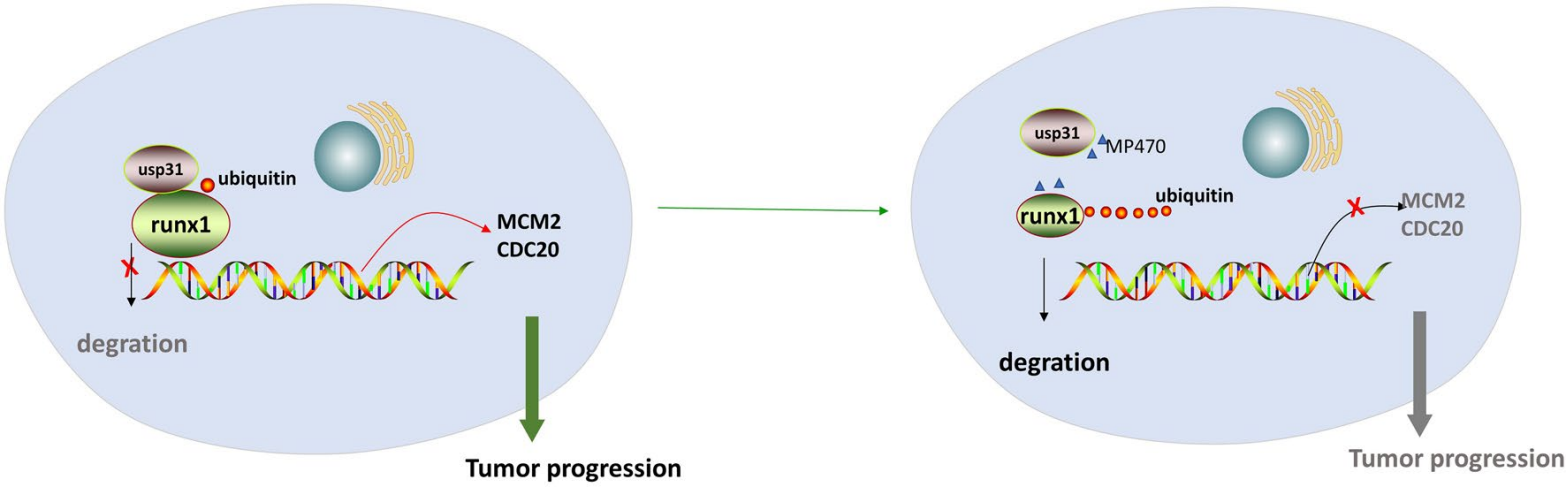
This schematic model depicts that RUNX1 is modified by usp31 deubiquitination to promote transcription of CDC20 and MCM2.

In this study, we investigated the role of RUNX1 in the progression of COAD. RUNX1 was strongly correlated with prognosis and could be used as a prognostic biomarker for COAD. RUNX1 was involved in growth and migration of COAD in vitro, and our results showed that MCM2 and CDC20 might be the target genes for RUNX1 transcription in COAD. In addition, there may be an interaction between the two proteins, RUNX1 and USP31, with modification of deubiquitination leading to upregulation of RUNX1 level expression (Fig. 7).

RUNX1 protein has been shown to have oncogenic or inhibitory effects by activating or suppressing target genes in a variety of cancers ^22,23^. In COAD, RUNX1 might have oncogenic function, and RUNX1 was reported to promote metastasis by activating Wnt/β-catenin, TGF-β and Hedgehog signaling pathways^20,24,25^. In the present study, we found that RUNX1 promoted the growth and migration of COAD. RUNX1 was overexpressed in COAD and its expression level increases with tumor progression. The expression of RUNX1 showed a negative correlation with the survival of patients. Furthermore, knockdown of RUNX1 resulted in a significant decrease in the proliferation and migratory capacity of COAD cells. Then the potential target genes of RUNX1 in COAD were further investigated. CHIP-qPCR analysis indicated that RUNX1 might bind to MCM2 and CDC20. MCM is an important initiator of DNA replication, which is necessary for DNA replication in all eukaryotic cells. There are 6 members of the MCM family proteins, which are MCM2~7. The hexamer complex formed by MCM2~7 loading forms the replication licensed complex of DNA, thus initiating DNA replication^26,27^. MCM2, as a member of its family, has very little MCM2 content in quiescent cells^28^. In proliferated and transformed cells, the content of MCM2 begins to increase in the G0 phase, reaches the peak at the end of the G0 phase and early s stage. It is free MCM2 from the s phase to the M phase and decreases in the G2 phase and M phase. Because of this periodic change consistent with the process of cell proliferation, it has been regarded as a marker of S-phase cells and a precancerous marker^29,30^. Furthermore, in previous studies, MCM2 has also been considered as a potential biomarker in colorectal cancer^31,32^. The expression of CDC20 also fluctuated with the change in the cell cycle. CDC20 could not be detected in the G1 phase of mitosis, appeared and accumulated in the late S phase, and reached the peak in the G2 M phase^33^. The binding of RUNX1 to CDC20 and MCM2 plays an important role in regulating the stability of mitotic cells. We speculate that the effect of RUNX1 on the prognosis of colorectal cancer as plays a transcriptional regulatory role, plays an important role in the occurrence and development of colorectal cancer depending on the binding of RUNX1 to mitotic regulatory proteins. In addition, we found that the expression of RUNX1, CDC20 and MCM2 correlated with the infiltration of tumor immune cells through bioinformatics analysis, and their expression may cause the tumor immune cells to be exhausted (Supplementary Fig. 5), and the possible mechanism of action remains to be investigated.

Our experimental results suggested that USP31 as a deubiquitinating enzyme is physically linked to RUNX1. Deubiquitin enzyme plays an important role in the process of cell proliferation and differentiation, and its functional changes are directly related to the occurrence and development of many cancers and neurodegenerative diseases^34–36^. USPs is the largest class of ubiquitin-removing enzymes, with 56 members, and is a large class of attractive and potential drug targets ^37^. USP31 as an important member of the deubiquitin enzyme, can affect the transcription of genes by regulating the NF-κB family^38,39^. MP470 was chosen as an inhibitor of the interaction between USP31 and RUNX1, results suggested that the USP31 protein acts through deubiquitination to affect the RUNX1 protein. The methylation level of USP31 was negatively correlated with the transcription level (Supplementary Fig. 6A) and its low methylation status in COAD may have contributed to the up-regulated expression (Supplementary Fig. 6B). Upregulated expression of USP31 mediates the stabilization and upregulation of RUNX1, which activates transcription of downstream genes in COAD.

In conclusion, in this study, we demonstrated that the RUNX1 transcription factor might act as an oncogene and a prognostic biomarker for COAD promotes cancer progression in COAD by activating target gene transcription. These discoveries might reveal the part RUNX1 plays in the development and spread of COAD, highlighting the therapeutic potential of focusing on RUNX1's oncogenic activity in COAD.

## Materials and Methods

### Data collection and identification of Differentially expressed genes (DEGs)

The COAD RNA-seq transcriptome profiles and related clinical information were downloaded from The Cancer Genome Atlas (TCGA) database (https://portal.gdc.cancer.gov/). The transcription factor (TF) set was downloaded from Cistrome Cancer (http://cistrome.org/CistromeCancer/). the gene expression was screened for subsequent analysis by the R software (version 4.1.2). The genes which meet the following criteria are defined as DEGs: the cut‐off value |log twofold‐change [FC]|> 1 and *p* value < 0.05.

### Prognostic signature construction

The least absolute shrinkage and selection operator (LASSO) analysis by using R software was performed to select optimal prognostic TFs. The multivariate Cox regression analysis was used to perform the risk model. To assess the performance of our signatures, the “survival receiver operating characteristic (ROC)” package was used to generate ROC curves at 1, 3 and 5 years, and the corresponding time-dependent area under the curves (AUCs) was calculated simultaneously. The performance of TFs-based signature was assessed using Kaplan–Meier analysis.

### Validation of the key genes based on TCGA

GEPIA was used to verify the survival and expression patterns.

### Cell culture

In DMEM with 10% fetal bovine serum (Procell) and added 1% (vol/vol) Penicillin–Streptomycin (Beyotime), HCT116 cells (Procell) and LOVO cells (American Type Culture Collection, ATCC) were kept at 37 °C in humidified air that contained 5% CO_2_.

### shRNA transfection

The short hairpin RNAs sequences (Supplementary Table 2) against RUNX1 (shRUNX1) and its corresponding nonspecific (NC) vectors were designed and constructed by Genechem Company (Shanghai, China). Lipofectamine 3000 transfection reagent (Thermo Fisher Scientific) was used to transfect cells with several plasmids in accordance with the owner's manual. Cells were harvested 24 h after transfection in preparation for the upcoming tests.

### Cell proliferation assays

After interfering with RUNX1 expression, HCT116 cells and LOVO cells (maintained by our laboratory) were inoculated into 96-well plates. Three replicate wells were set up for each group, and cell proliferation was detected with the Cell Counting Kit 8 (CCK-8).

### Colony formation

HCT116 cells and LOVO cells were seeded into 6-well plates with 1000 cells per well after interfering with RUNX1 expression and cultured in medium containing 10% FBS for 10 days. The colonies were fixed with 4% paraformaldehyde for 20 min and stained with crystal violet solution for 5 min. The number of colonies was calculated.

### Transwell migration assay

Plated 5 × 10^4^ cells in triplicate in a migration chamber using serum-free medium. Cells were given 48 h to move through Transwells into medium containing 10% FBS. Cells that did not migrate through the membrane were eliminated, and those that did migrate were fixed with 4% paraformaldehyde. The fixed cells were then stained and counted.

### Transcriptome sequencing and differentially expressed genes analysis

Total RNA was extracted from HCT116 cells, whole transcriptome sequencing and bioinformatics data were analyzed at Novogene (Beijing, China). The differentially expressed genes (DEGs) from the RNA sequencing results were analyzed after transcriptome sequencing. Predicting target genes by using the hTFtarget database (http://bioinfo.life.hust.edu.cn/hTFtarget#!/).

### Enrichment analysis

Gene Ontology (GO) and Kyoto Encyclopedia of Genes and Genomes (KEGG) pathway enrichment analyses of the DEGs were performed using the “ClusterProfiler” R package.

### Quantitative PCR

The Trizol reagent (Thermo Fisher Scientific) was used to total RNA isolation of HCT116 cells. Using the Superscript III First Strand Synthesis System, the RNA was reverse transcribed into complementary DNA (Thermo Fisher Scientific). The SYBR green PCR Master Mix (Thermo Fisher Scientific) were used to measure the amount of gene's expression. Using GAPDH as a normalization control, the expression of each target gene was assessed. The analysis yielded the Ct values for the amplified products, and the data were evaluated using the 2^−ΔΔCt^ technique. The primers were listed in Supplementary Table 3.

### Chromatin immunoprecipitation analysis

Cells were collected and treated with 1% formaldehyde to cross-link chromatin-associated proteins to DNA. DNA was digested with micrococcal nuclease (NEB, Ipswich, MA). Ten percent of each sample was used as an input reference control. Chromatin protein suspensions were then incubated with anti-RUNX1 antibody (Proteintech, 25315-1-AP) and negative control IgG antibody (Proteintech, 30000-0-AP) overnight at 4 °C. All the above chromatin supernatants were incubated with magnetic protein A/G (MCE) beads at temperature for 2 h. The protein-DNA complexes were inverted and purified to pure DNA and then subjected to qPCR analysis. The primers were listed in Supplementary Table 3.

### Western blotting

To prepare total protein, the cells were lysed in RIPA buffer supplemented with 1% PMSF on ice. Extracted protein samples were analyzed through 12.5% sodium dodecyl sulfate–polyacrylamide gel electrophoresis (SDS-PAGE) and were electro-transferred onto the polyvinylidene difluoride (PVDF) membranes. Next, the membranes were blocked with 4% blocking solution prepared by TBST and bovine serum albumin for 0.5 h on a shaking table. After blocking, the membranes were incubated with specific primary antibodies at 4℃ overnight. Membranes were washed with TBST and then incubated with 1:2000 secondary antibodies for 1 h, and developed with enhanced chemiluminescence (ECL).

### Co-Immunoprecipitation (Co-IP) analysis

Cells were collected, rinsed twice with pre-chilled PBS, and then lysed for 30 min on ice in western and RIPA buffer (Beyotime) containing protease inhibitor cocktail (Beyotime). Co-IP experiments were performed with anti-RUNX1 antibody (Proteintech, 25315-1-AP), anti-USP31 (Proteintech, 12076-1-AP) antibody and negative control IgG antibody. Finally, the Protein A/G Magnetic beads (MCE, USA) were washed twice with Western and RIPA buffer, and the proteins were eluted by boiling at 95 °C for 5 min in 1 × loading buffer and then used for Western blotting.

### Ubiquitination analysis

HCT116 cells was treated with MP470 (0.1 µM, 24 h, AbMole), then the cells in the control and treated groups were incubated with 10 μM MG132 (Beyotime) for 6 h, immunoprecipitation was then performed using an anti-RUNX1 antibody (Proteintech, 25315-1-AP). The immunoprecipitated proteins were subjected to western blot to evaluate the ubiquitination level with ubiquitin antibody (Proteintech, 10201-2-AP).

### Obtaining inhibitors of RUNX1 and USP31

The drug sensitivity analysis tool in the online tool GSCA: Gene Set Cancer Analysis (https://guolab.wchscu.cn/GSCA/#/drug) was used to find the inhibitors of RUNX1 and USP31 in GDSC.

### Molecular docking

To analyze the binding affinities between the drug candidate and their targets, AutodockVina1.2.2 software (http://autodock.scripps.edu/) was employed. Molecular structures of FH535 and MP470 were retrieved from PubChem Compound (https://pubchem.ncbi.nlm.nih.gov/). 3D coordinates of RUNX1 (PDB ID, 1E50; resolution, 2.60 Å) was downloaded in 3D coordinates from PDB (http://www.rcsb.org/pdb/home/home.do). 3D coordinates of USP31 (Predicted; Identifier, AF-Q86UV5-F1) was downloaded in 3D coordinates from UniProt (https://www.uniprot.org/). For visualization, docking results were analyzed using PyMol software (https://pymol.org/2/).

### Statistical analysis

The GraphPad Prism (version 7.01) statistical analysis software programs were used for statistical analysis of the experimental data. Each experiment was repeated at least three times. Data were presented as the mean ± SD and analyzed using Student’s t test or one-way ANOVA. P value less than 0.05 was considered statistically significant.

### Supplementary Information

Supplementary Information.

## Data Availability

Publicly available datasets were analyzed in this study, these can be found in The Cancer Genome Atlas (https://portal.gdc.cancer.gov/) and the Cistrome Cancer (http://cistrome.org/CistromeCancer/).
