# Supplementary material for: RUNX1 regulates MCM2/CDC20 to promote COAD progression modified by deubiquitination of USP31
Source: Sci Rep. 2024 Jun 17;14:13906. doi: 10.1038/s41598-024-64726-w (PMC11183096; doi:10.1038/s41598-024-64726-w)
Supplement: Supplementary file 1 — Supplementary Information. [file 41598_2024_64726_MOESM1_ESM.docx]

**RUNX1 regulates MCM2/CDC20 to promote COAD progression modified by deubiquitination of USP31**

Wei Tian^1,4#^, Jingyuan Zhao^2#^, Xinyu Zhang^1,4#^, Pengfei Li^1,4^, Xuening Li^4^, Yuan Hong^3*^, Shuai Li^5*^


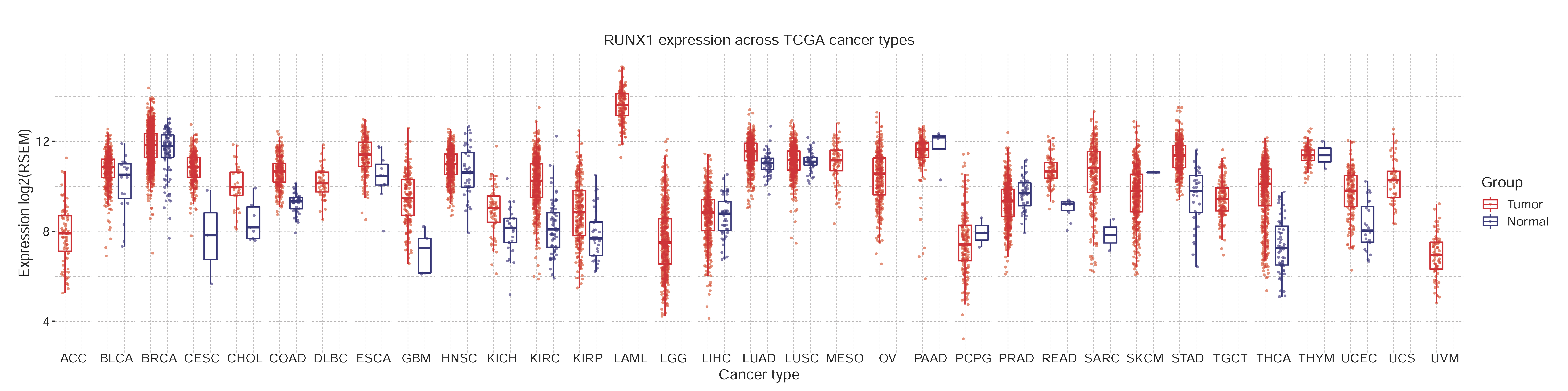


Supplementary FIGURE 1| Expression of RUNX1 in pan-cancer.


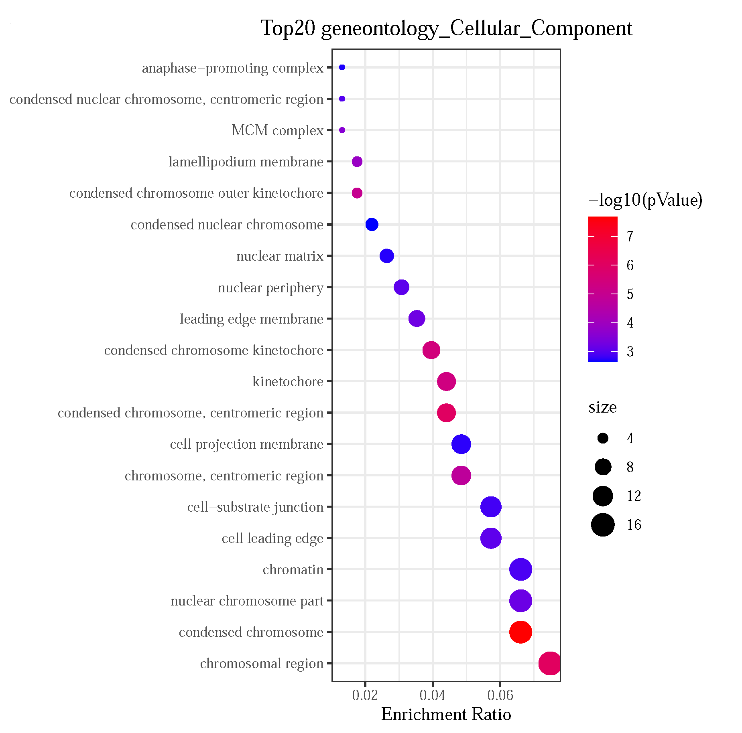


Supplementary FIGURE 2| Significantly enriched GO cellular component of the above obtained target genes.


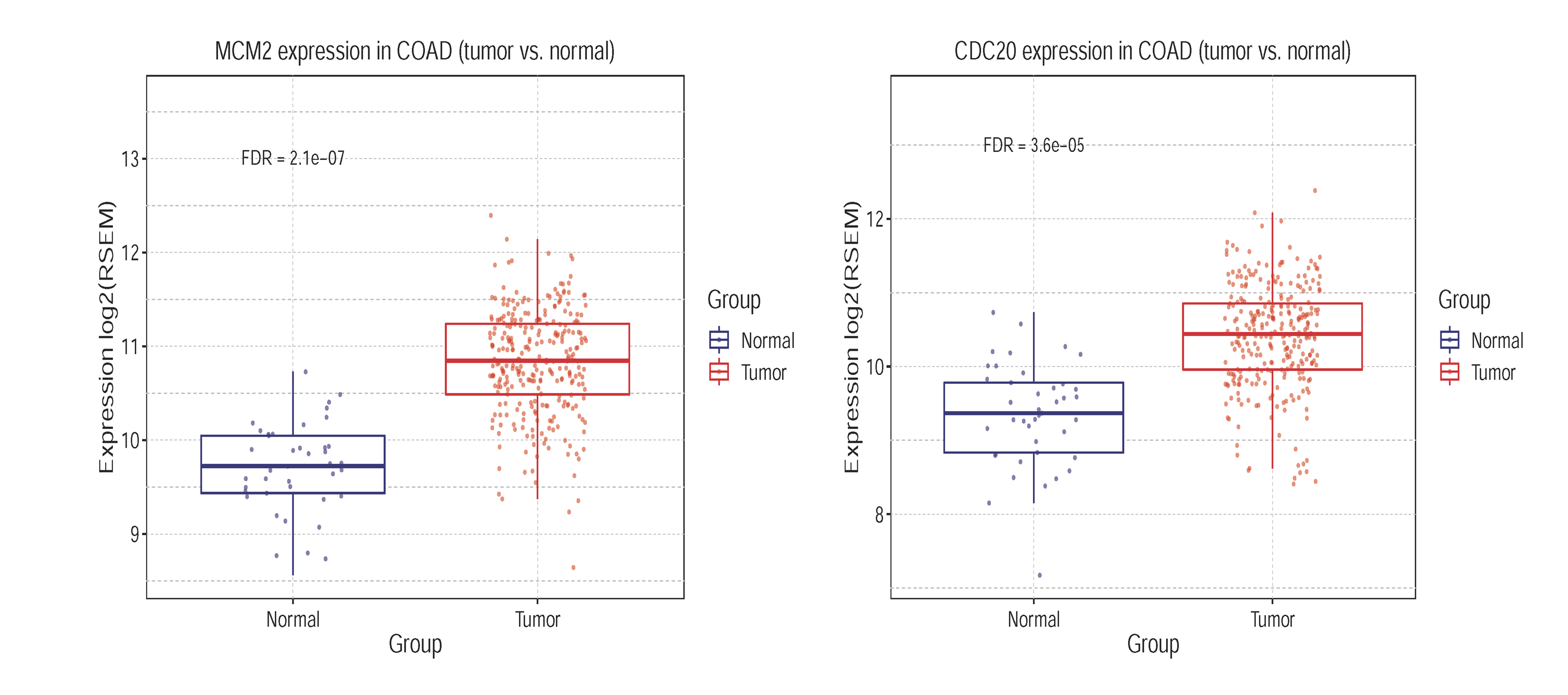


Supplementary FIGURE 3| Expression of MCM2 and CDC20 in COAD.


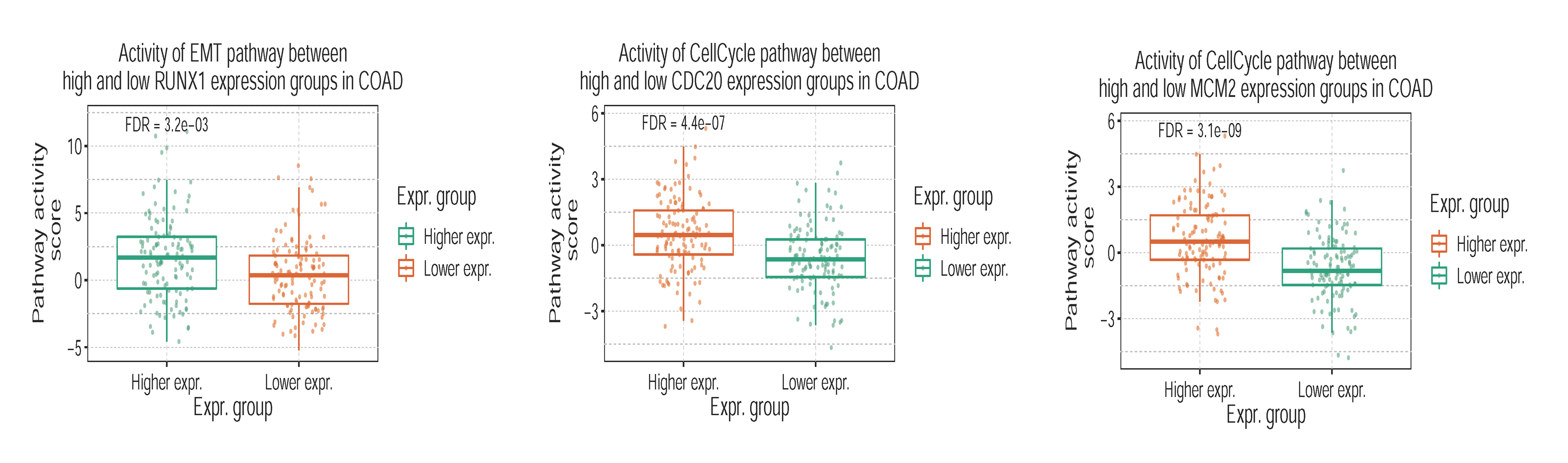
 Supplementary FIGURE 4| Correlation of MCM2 and CDC20 expression with pathways in COAD.


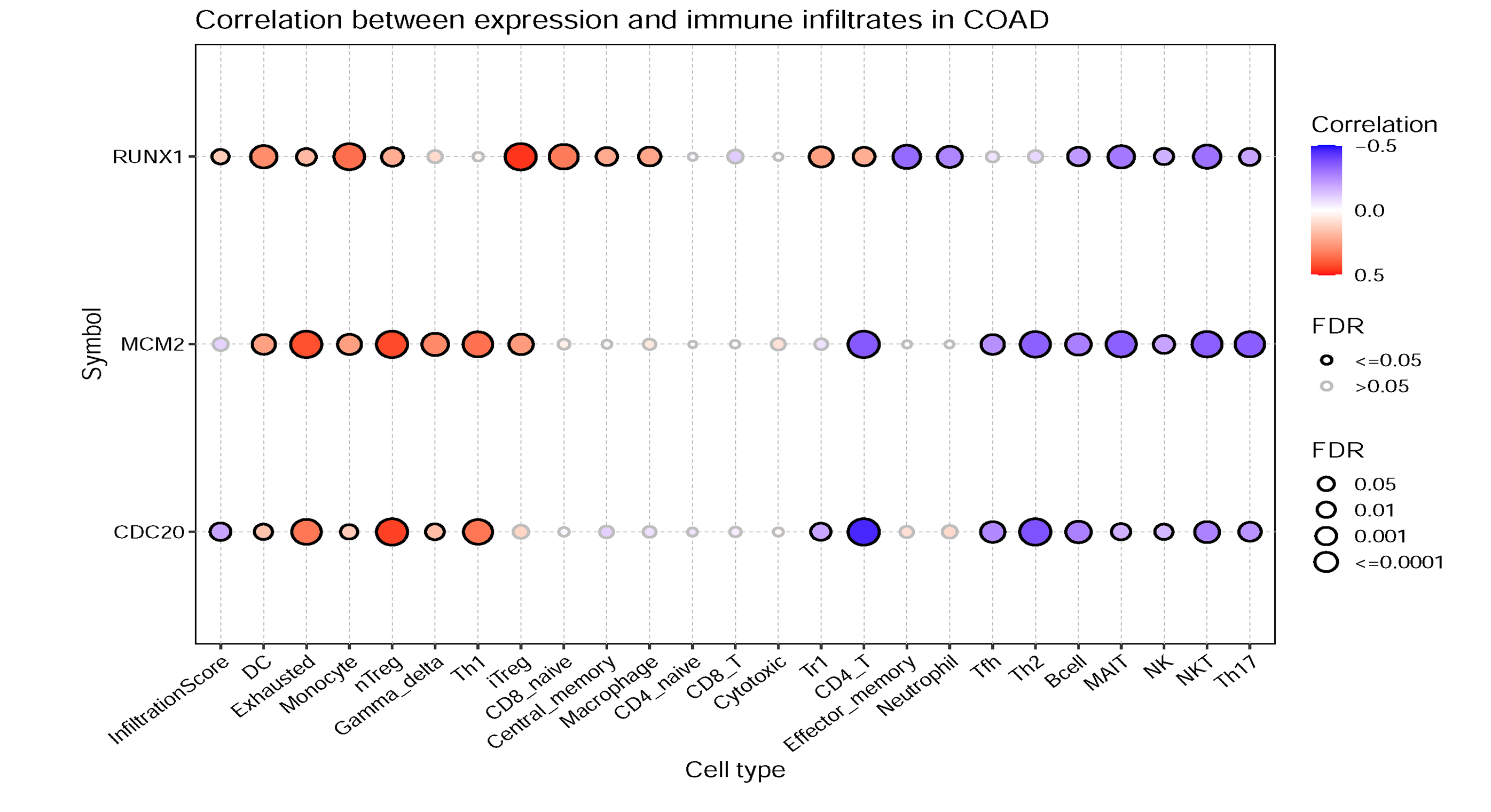


Supplementary FIGURE 5| The correlation between genes and tumor-infiltrating immune cell type.


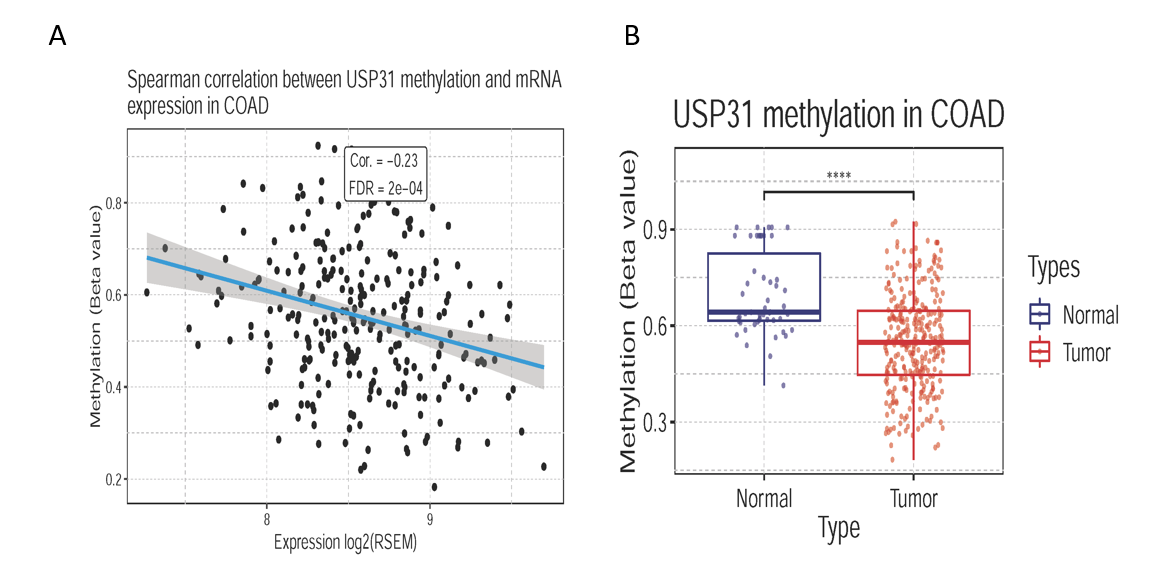


Supplementary FIGURE 6 | The relationship between USP31 methylation and transcription level in TCGA COAD dataset.

Supplementary TABLE 1 | Binding energy for targets with their inhibitors

| Target | Inhibitor | Binding Energy (kcal/mol) |
| --- | --- | --- |
| RUNX1 | FH535 | -4.548 |
| RUNX1 | FH535 | -4.466 |
| RUNX1 | FH535 | -4.463 |
| RUNX1 | FH535 | -4.443 |
| RUNX1 | FH535 | -4.44 |
| RUNX1 | FH535 | -4.38 |
| RUNX1 | FH535 | -4.375 |
| RUNX1 | FH535 | -4.341 |
| RUNX1 | FH535 | -4.298 |
| USP31 | FH535 | -7.281 |
| USP31 | FH535 | -6.603 |
| USP31 | FH535 | -6.555 |
| USP31 | FH535 | -6.518 |
| USP31 | FH535 | -6.417 |
| USP31 | FH535 | -6.384 |
| USP31 | FH535 | -6.336 |
| USP31 | FH535 | -6.327 |
| USP31 | FH535 | -6.321 |
| RUNX1 | MP470 | -6.103 |
| RUNX1 | MP470 | -6.091 |
| RUNX1 | MP470 | -5.995 |
| RUNX1 | MP470 | -5.955 |
| RUNX1 | MP470 | -5.905 |
| RUNX1 | MP470 | -5.853 |
| RUNX1 | MP470 | -5.699 |
| RUNX1 | MP470 | -5.698 |
| RUNX1 | MP470 | -5.679 |
| USP31 | MP470 | -8.651 |
| USP31 | MP470 | -8.402 |
| USP31 | MP470 | -8.278 |
| USP31 | MP470 | -8.276 |
| USP31 | MP470 | -8.267 |
| USP31 | MP470 | -8.26 |
| USP31 | MP470 | -8.192 |
| USP31 | MP470 | -8.059 |
| USP31 | MP470 | -8.002 |

Supplementary TABLE 2 | shRUNX1 sequences

| Target | sequences |
| --- | --- |
| shRUNX1-1 | GGATACAAGGCAGATCCAACC |
| shRUNX1-2 | GCTGAGCTGAGAAATGCTACC |

Supplementary TABLE 3 | The primers of genes

| Target | sequences |
| --- | --- |
| PKMYT1-F | 5’-CATGGCTCCTACGGAGAGGT-3’ |
| PKMYT1-R | 5’-ACATGGAACGCTTTACCGCAT-3’ |
| CDC25C-F | 5’-TCTACGGAACTCTTCTCATCCAC-3’ |
| CDC25C-R | 5’-TCCAGGAGCAGGTTTAACATTTT-3’ |
| MCM2-F | 5’-ATGGCGGAATCATCGGAATCC-3’ |
| MCM2-R | 5’-GGTGAGGGCATCAGTACGC-3’ |
| CDC25A-F | 5’-GTGAAGGCGCTATTTGGCG-3’ |
| CDC25A-R | 5’-TGGTTGCTCATAATCACTGCC-3’ |
| CDC20-F | 5’-GCACAGTTCGCGTTCGAGA-3’ |
| CDC20-R | 5’-CTGGATTTGCCAGGAGTTCGG-3’ |
| GAPDH-F | 5’-ACAACTTTGGTATCGTGGAAGG-3’ |
| GAPDH-R | 5’-GCCATCACGCCACAGTTTC-3’ |
| RUNX1-F | 5’-CTGCCCATCGCTTTCAAGGT-3’ |
| RUNX1-R | 5’-GCCGAGTAGTTTTCATCATTGCC-3’ |
| N-cadherin-F | 5’-TGCGGTACAGTGTAACTGGG-3’ |
| N-cadherin-R | 5’-GAAACCGGGCTATCTGCTCG-3’ |
| Vimentin-F | 5’-AGTCCACTGAGTACCGGAGAC-3’ |
| Vimentin-R | 5’-CATTTCACGCATCTGGCGTTC-3’ |
| SNAI1-F | 5’-TCGGAAGCCTAACTACAGCGA-3’ |
| SNAI1-R | 5’-AGATGAGCATTGGCAGCGAG-3’ |
| SNAI2-F | 5’-CGAACTGGACACACATACAGTG-3’ |
| SNAI2-R | 5’-CTGAGGATCTCTGGTTGTGGT-3’ |


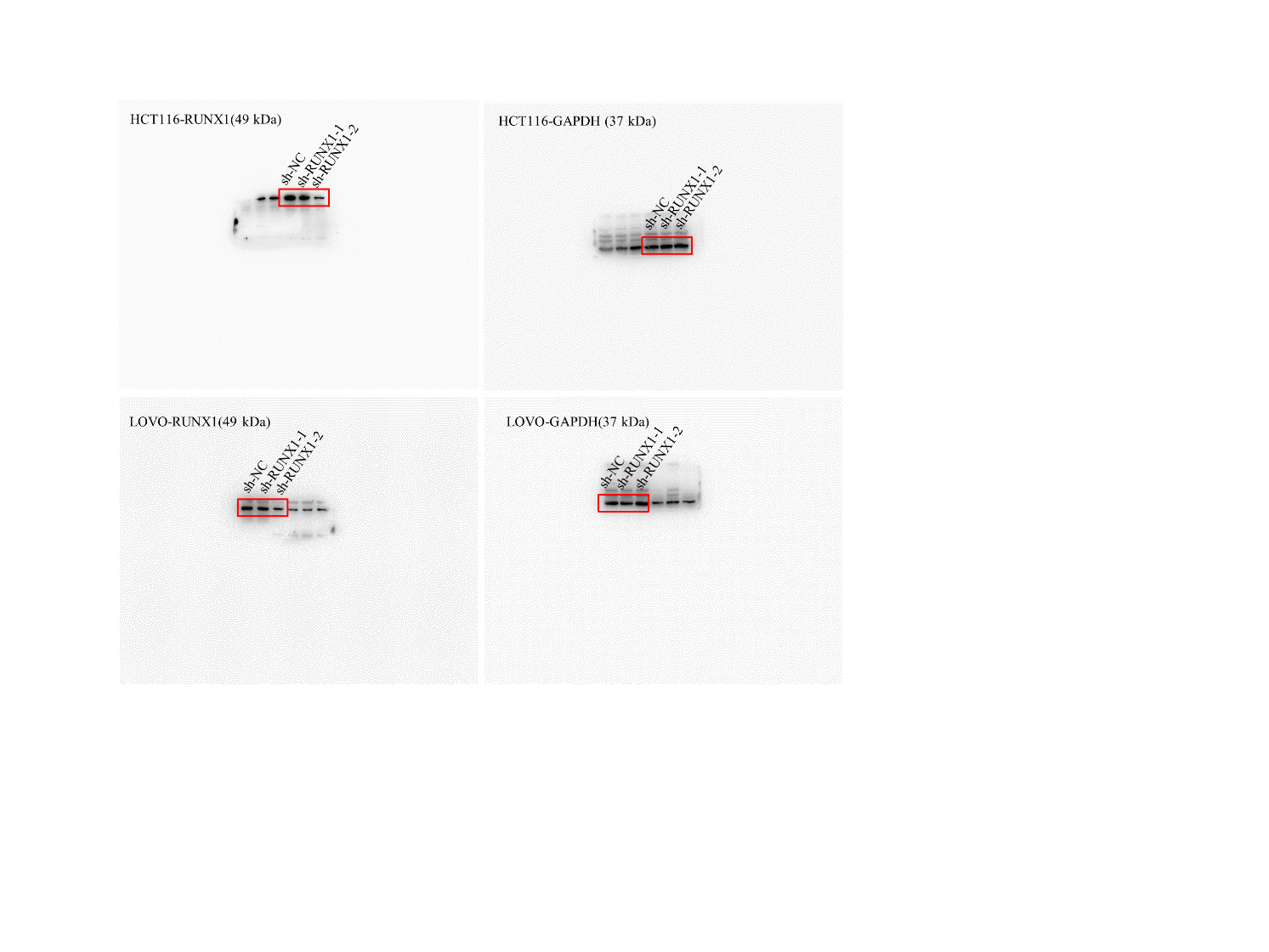


Supplementary FIGURE 7 | Levels of RUNX1 protein in HCT116 and LOVO cells transfected with a plasmid encoding shRUNX1 were analyzed by Western blotting.


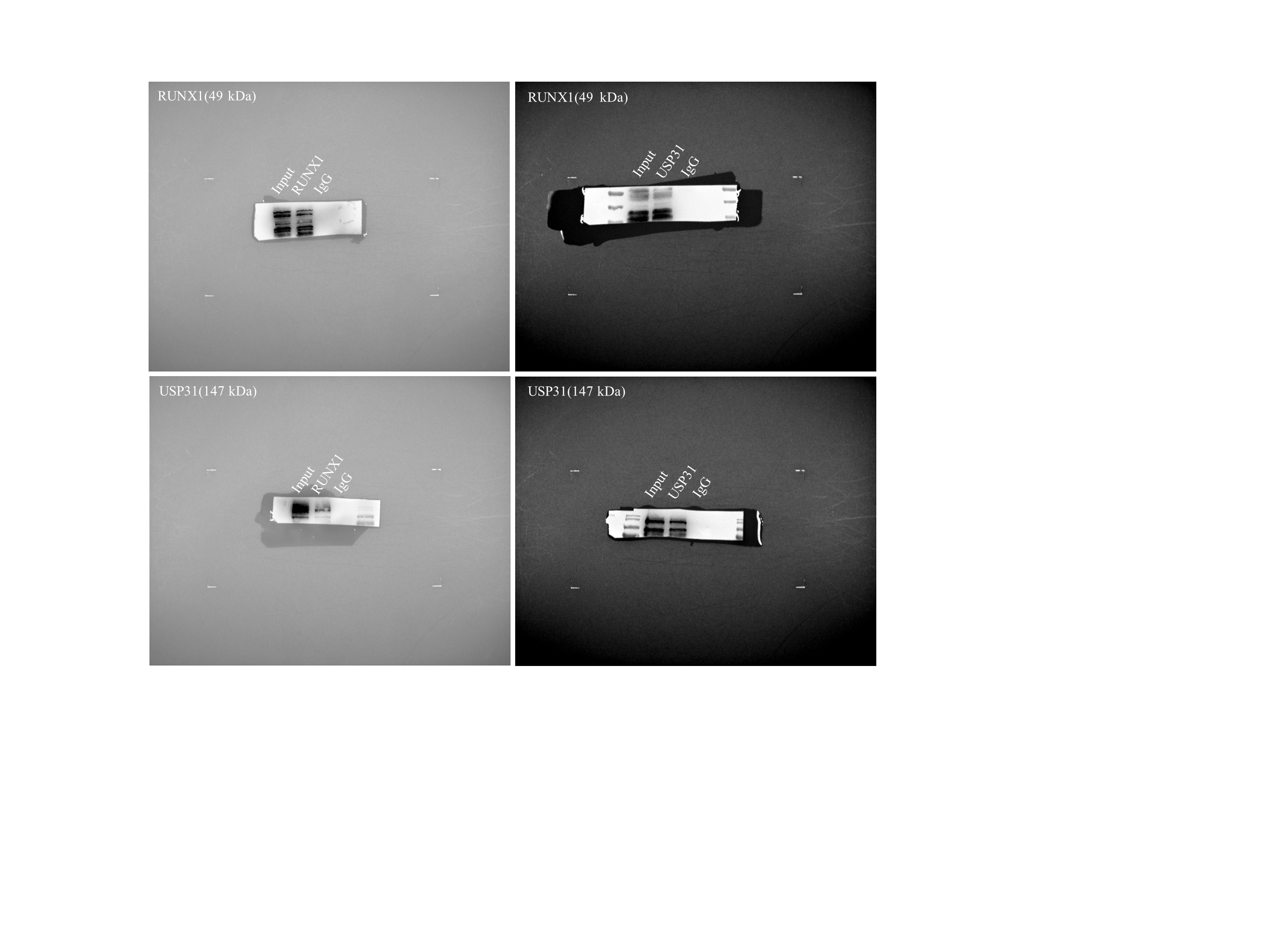


Supplementary FIGURE 8 | Coimmunoprecipitation (co-IP) of RUNX1 and USP31 from HCT116 cells. IgG was used as the control.


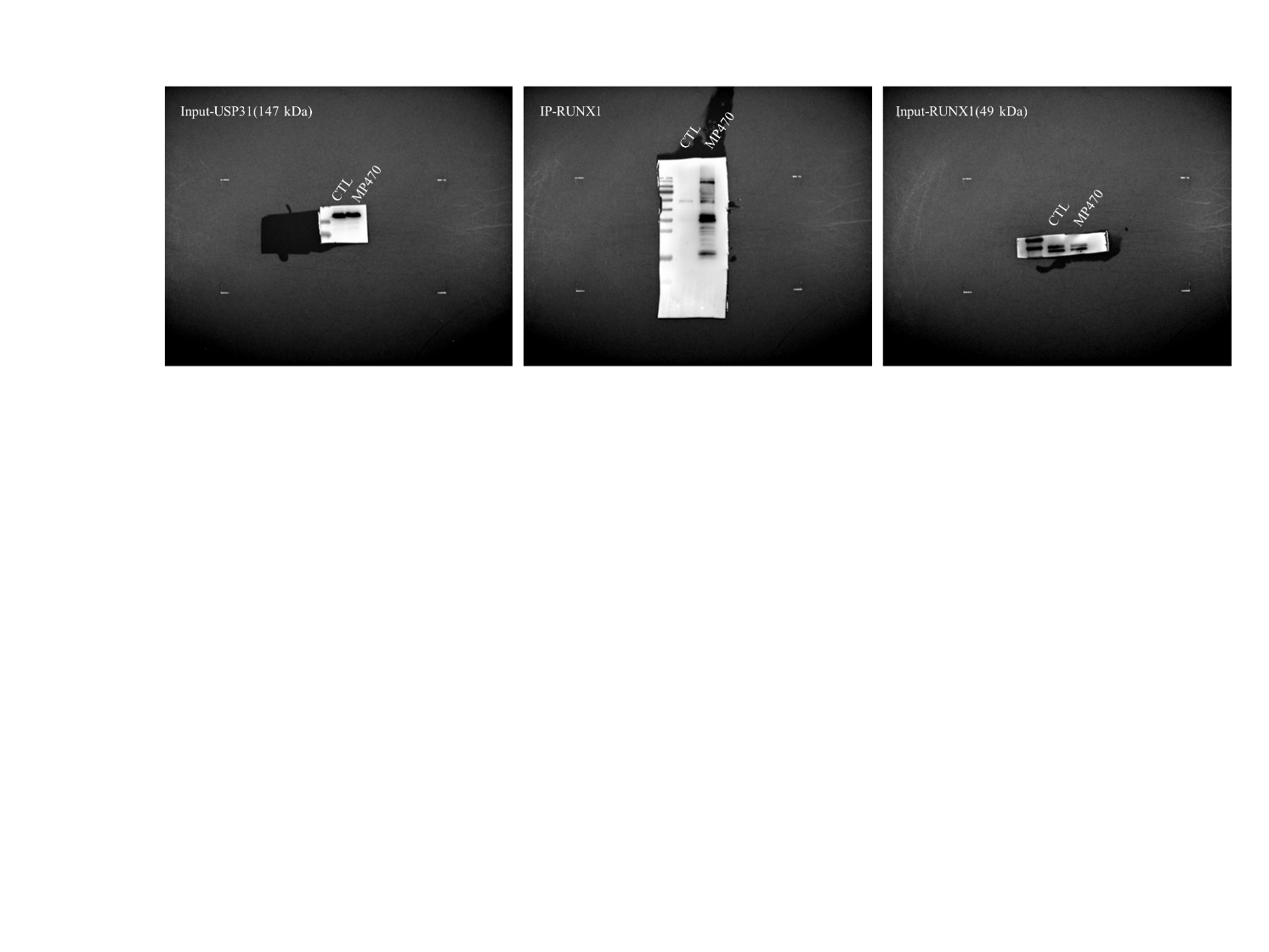


Supplementary FIGURE 9 | Co-IP from untreated and MP470-treated HCT116 cells. IgG was used as the control.
